# Using point-of-sale data to examine tobacco pricing across neighbourhoods in Scotland

**DOI:** 10.1136/tobaccocontrol-2019-055484

**Published:** 2020-03-19

**Authors:** Niamh K Shortt, Helena Tunstall, Richard Mitchell, Emma Coombes, Andy Jones, Garth Reid, Jamie Pearce

**Affiliations:** 1 School of Geosciences, Univerity of Edinburgh, Edinburgh, UK; 2 MRC/CSO Social and Public Health Sciences Unit, University of Glasgow, Glasgow, UK; 3 Norwich Medical School, University of East Anglia, Norwich, UK; 4 NHS Health Scotland, Edinburgh, UK

**Keywords:** availability, price, deprivation, outlet density, sales data

## Abstract

**Objectives:**

To assess the geographical variation in tobacco price (cigarettes and roll-your-own (RYO) tobacco) in convenience stores across Scotland and how this relates to neighbourhood income deprivation, tobacco retail outlet density and urban/rural status.

**Methods:**

Tobacco price data from 124 566 shopping baskets purchased in 274 convenience stores during 1 week in April 2018 were obtained through an electronic point-of-sale system. These data were combined with neighbourhood-level measures of income deprivation, tobacco retail outlet density and urban/rural status. We examined brand price for 12 of the most popular cigarette brands and 3 RYO brands and variations in purchases by price segment; multivariable regression analysis assessed associations between area variables and tobacco price.

**Results:**

Most stores sold tobacco in all price segments. The lowest priced subvalue brands were the most popular in all neighbourhoods but were most dominant in shops in more deprived neighbourhoods. When total sales were assessed, overall purchase price varied significantly by neighbourhood income deprivation; packets of 20 cigarettes were 50 pence (5.6%) lower and RYO 34 pence (2.7%) lower among shops in the two highest income deprivation quintiles relative to the lowest. Analysis of individual brands showed that for 3 of the 12 cigarette brands considered, average prices were 12–17 pence lower in more deprived neighbourhoods with the most popular RYO brand 15 pence lower. There was limited evidence of a relationship with tobacco retail outlet density.

**Conclusion:**

Across Scottish convenience stores, the purchase price of cigarettes and RYO was lower in more income-deprived neighbourhoods. The lower prices primarily reflect greater sales of cheap brands in these areas, rather than retailers reducing the prices of individual brands.

## Introduction

Research has demonstrated that tobacco price is one of the most important determinants of smoking behaviour.^1–4^ Although smoking rates have been in long-term decline in high-income countries,^5^ substantial socioeconomic inequalities in smoking persist.^6^ Recent years have seen the increasing price segmentation of tobacco products,^7^ the rise of cheap tobacco brands^8^ and the tobacco industry’s policy of ‘undershifting’ taxation (increasing the price of premium brands to absorb tax rises on the cheapest brands) to limit the impact of tax rises^9 10^ All these factors pose challenges to achieving the ‘Tobacco Endgame’ of reducing tobacco use and/or smoking prevalence to less than 5%.^11^

Neighbourhood differentials in tobacco price are potentially important in understanding geographical variations in smoking rates.^12–15^ Socioeconomic status of neighbourhoods, retail density and urban/rural differences have all been suggested as drivers of tobacco price. Research has found lower average price paid for cigarette products^16^ and lower prices for individual brands^17^ in low-income neighbourhoods. Previous research has also shown that tobacco retail density is related to smoking rates,^18^ to young people trying smoking^19^ and to reduced success in quitting.^20^ Price difference is one potential pathway between retail density and smoking behaviours; a greater concentration of tobacco retail outlets may lead to greater competition and lower prices. However, the evidence for this is mixed, with some analysis finding reduced distance between tobacco retailers to be associated with lower prices for the cheapest cigarettes and certain brands^13^ but most studies of outlet density finding no relationship with price.^13 15 21^ Finally, tobacco price analysis has also explored urban/rural differences, suggesting that cigarettes are more expensive in urban areas with higher population densities.^22–24^

While this body of research indicates that price may vary by neighbourhood-level characteristics, the methodology used to collect pricing data has limited the quality of the evidence to date. Most studies have assessed tobacco prices either through self-report surveys^23^ or by visiting stores and recording sale price of a small number of brands.^16 22 24^ The labour-intensive nature of this data collection process has precluded geographical work examining price segmentation or the price distribution of all tobacco purchased within neighbourhoods.

In recent years, there has been increasing interest in innovative approaches to tobacco pricing.^25^ In Scotland, minimum unit pricing (MUP) of tobacco has been suggested as a potential new approach for tobacco control.^26^ Any such moves towards MUP for tobacco will require a more detailed understanding of purchase price and the potential drivers of price, including neighbourhood characteristics. The prices at which tobacco brands are sold by retailers in the UK, including Scotland, can vary by store. While tobacco companies emphasise the importance of the recommended retail price (RRP), individual retailers can increase sales prices above the RRP to increase profit margins or lower prices to attract price sensitive customers.^27^ In response, this study explores neighbourhood-level variation in the purchase price of tobacco products in Scotland, both cigarettes and roll your own tobacco. In a novel approach, we have gathered a point-of-sale dataset that captures all tobacco sales over a 1 week period across 274 individual stores in Scotland. We examine how brand purchase price varies by neighbourhood-level income deprivation, tobacco retail availability and urban/rural status. We believe this is the first study to report on neighbourhood variation for all tobacco sales, not just the most popular or cheapest brands.

## Methods

Tobacco price data describing purchases of tobacco products from a group of convenience stores across Scotland were provided by The Retail Data Partnership (TRDP). In the UK, convenience stores are smaller stores selling a variety of grocery items, including tobacco products. These stores are the most common type of tobacco retail outlets in Scotland, accounting for 37.5% of the total 9042 tobacco outlets registered in 2016 and 55%–60% of total tobacco sales.^28^ TRDP supply an electronic point-of-sale until to 275 convenience stores in Scotland, capturing information on every item purchased within these outlets. TRDP provided a data extract on all products in ‘tobacco baskets’ (ie, baskets containing one or more tobacco, smoking accessory or e-cigarette vaping products) purchased over a 7 day period (16–22 April 2018). One store with very low sales (<50 products in tobacco baskets in the study week) was excluded, leaving 274 shops in the analysis.

The focus of this research was on the prices of factory made packets of 20 cigarettes and 30 g of roll your own (RYO) tobacco. Each brand was allocated into a price segment category based on a combination of the categorisation used by Hiscock *et al*,^9^ recent wholesaler recommended sales price (RSP) and sales prices. For cigarettes, these categories were subvalue, value, midprice and premium. For roll-your-own (RYO) cigarettes, the categories were value, midprice and premium, later combined to value and midprice/premium due to a lack of distinction between sales prices within the latter two categories. For cigarette and RYO 30 g brand variants, 4.3% and 16.7% of brands respectively, were not classified within a price segment because information from Hiscock *et al*,^9^ wholesaler RSP and shop prices was absent or inconsistent. In our sample, they accounted for very low sales, 0.0005% of total cigarette 20 pack sales and 0.205% of total RYO 30 g pack sales.

Neighbourhood-level variables of income deprivation, tobacco retail outlet density and urban/rural status were linked to each store by the TRDP. Neighbourhood income deprivation was derived from the Scottish Index of Multiple Deprivation 2016 Income Domain. This measure is based on the prevalence of welfare benefits, for which low income dictates eligibility.^29^ Rural/urban status was derived from the Scottish Government 8-fold Urban Rural Classification 2016. Each were defined for Data Zones, small areas with a population of around 500–1000 residents. Stores were grouped into income deprivation quintiles, containing equal numbers of stores, based on their Data Zone’s income deprivation rank. Using the Scottish Government’s Urban Rural Classification of neighbourhoods, the Data Zone of each shop was defined as located within one of four settlement types: large urban areas (populations ≥125 000), other urban areas (population 10 000–124 999), small towns (population 3000–9999) and rural areas (population <3000).

Tobacco retail outlet density was a measure of the density of tobacco retailing for each postcode across Scotland. It was calculated using data from the Scottish Tobacco Retailers Register in 2016, which includes locational data on all outlets selling tobacco in Scotland (n=9042). This information was used to calculate a Kernel Density Estimation (KDE) value for each postcode (each postcode represents approximately 15 address points) in Scotland. The KDE process divides Scotland into 100×100 m grid cells and assesses the number and proximity of outlets within an 800 m radius of each cell. Outlets near the centre of the search window are given greater weight than those further away (see Shortt *et al* 30). The final KDE values represent the density of tobacco outlets per km^2^ rather than a count of tobacco outlets per area. The tobacco outlet density cell values of each shop’s postcode were then used to categorise shops into five outlet density quintiles containing equal numbers of shops based on density rank.

The location of 274 TRDP stores in the study was compared with convenience stores across Scotland using data from the 2016 Scottish Tobacco Retailers Register. This indicated that convenience stores in the study relative to those found nationwide were more likely to be in deprived areas but had similar locations in respect of outlet density and urban/rural status.

Relationships between the area-level variables, income deprivation, outlet density and urban/rural status and tobacco price and were assessed using linear regression models for the analysis of all sales, the lowest priced tobacco brand sold and individual brand prices. Binary logistic regression models were used to analyse the relationship between the area level variables and the odds that tobacco products sold were in particular price segments. Models of all sales and price segment (both at the individual product level) were adjusted for the clustering of sales at shop level to correct SEs. Relationships were judged to be significant when there was p<0.05. All analysis was completed using Stata/SE V.14.2.

## Results

There were 124 566 baskets containing tobacco leaf, smoking accessory or e-cigarette products purchased during the study week (online supplementary table S1). Among the 412 761 items in these baskets, 30.0% were tobacco leaf products, 6.6% smoking accessories and 0.6% e-cigarettes, with the remaining items in the basket non-tobacco related. The most common tobacco leaf purchases were cigarette 20 packs (89.5%), followed by RYO 30 g (7.5%), RYO 50 g (1.2%), cigars (1.0%), pipe tobacco (0.5%) and cigarette packs larger than 20 (0.4%). Further analysis is restricted to cigarette 20 packs and RYO 30 g.

10.1136/tobaccocontrol-2019-055484.supp1 Supplementary data

### The prices of cigarettes and RYO products purchased

For all packets of 20 cigarettes sold in the study week, the average price was £8.49 (table 1), with prices ranging from £7.29 to £13.25. For RYO 30 g, the average price paid was £12.14, with prices ranging from £9.80 to £15.99 (see table 2 for examples of price ranges).

**Table 1:** Summary descriptives: cigarettes and RYO in tobacco baskets by area type

Area type	Shops	Cigarette packets of 20								RYO 30 g					
	Total	Mean shop sales	Mean sales price	Mean shop lowest priced brand variant	Mean shop number of brand variants sold	Percentage of brand variants sold in price segment*				Mean shop sales	Mean sales price	Mean shop lowest priced brand variant	Mean shop number of brand variants sold	Percentage of brand variants sold in price segment*	
						Subvalue	Value	Midprice	Premium					Value	Mid price/premium
	N	N	£	£	N	%	%	%	%	N	£	£	N	%	%
Total – all areas	274	404.5	8.49	7.73	40.9	51.8	28.9	14.4	5.0	33.8	12.14	10.57	6.8	44.5	55.3
SIMD income deprivation															
1 lowest deprivation	55	271.9	8.89	7.77	37.5	38.6	32.9	17.4	11.1	23.2	12.42	10.73	6.4	37.0	62.5
2	55	299.2	8.63	7.71	35.1	43.4	32.3	17.3	7.0	26.1	12.37	10.64	6.1	35.2	64.5
3	55	432.2	8.46	7.69	43.0	50.0	30.3	15.1	4.6	38.2	12.20	10.56	7.0	39.2	60.5
4	55	481.0	8.30	7.73	42.6	58.8	26.2	11.9	3.1	38.1	11.88	10.53	7.2	52.6	47.3
5 highest deprivation	54	540.6	8.38	7.74	46.4	58.3	26.2	12.8	2.7	43.4	12.04	10.39	7.7	51.9	48.1
Tobacco outlet density															
1 lowest density	54	305.3	8.59	7.76	32.8	45.5	31.7	16.6	6.2	25.2	12.24	10.81	5.6	38.4	61.3
2	55	460.7	8.39	7.69	40.5	54.8	27.8	13.7	3.7	34.4	12.02	10.50	6.7	46.3	53.6
3	55	424.1	8.44	7.73	40.6	52.5	28.8	14.3	4.5	32.5	12.21	10.59	6.4	43.0	56.8
4	55	430.7	8.44	7.69	44.7	53.5	27.7	14.0	4.8	34.2	12.05	10.44	7.1	45.9	54.0
5 highest density	55	399.9	8.61	7.78	45.8	50.5	29.5	13.9	6.1	42.2	12.21	10.51	8.3	46.7	52.9
Urban/rural status															
Large urban areas	103	448.8	8.54	7.74	47.4	50.8	29.4	14.1	5.6	36.4	12.15	10.53	7.8	46.4	53.3
Other urban areas	95	446.3	8.41	7.72	40.7	55.0	27.8	13.5	3.7	37.0	12.05	10.48	6.7	48.6	51.3
Small towns	24	383.2	8.48	7.69	38.9	48.0	31.1	16.1	4.8	36.8	12.29	10.62	6.8	34.2	65.8
Rural areas	52	250.1	8.54	7.74	29.3	47.3	29.3	16.8	6.6	21.1	12.30	10.79	5.1	33.5	66.1

*Percentages do not sum to 100% because the price segment of some brands was not classified.

RYO, roll your own; SIMD, Scottish Index of Multiple Deprivation.

**Table 2:** Best-selling packet of 20 cigarette and RYO 30 g brand variant descriptives

Rank	Brand variant name	Price segment	Number of sales (N)	Proportion of total sales (%)	Proportion of total shops selling (%)	Wholesaler RSP April/September* 2018 (£)	Mean of shop prices (£)	Min. price (£)	Max. price (£)	Range of prices (£)
Cigarette packets of 20										
1	Players Kingsize Real Red	Subvalue	14 766	13.3	97.1	7.65	7.88	7.55	9.65	2.10
2	Players Superking Real Red	Subvalue	13 496	12.2	94.9	7.65	7.88	7.55	9.85	2.30
3	L&B Kingsize Blue Real Blue	Value	5708	5.2	80.3	8.00	8.22	7.75	10.25	2.50
4	Kensitas Club* Kingsize	Subvalue	5701	5.1	64.6	10.55	7.84	7.50	10.65	3.15
5	JPS Kingsize Real Blue	Value	5074	4.6	94.9	8.30	8.59	7.85	10.49	2.64
6	L&B Kingsize Original Silver	Midprice	4628	4.2	93.1	9.30	9.64	7.65	11.65	4.00
7	Mayfair Kingsize Original Blue	Midprice	4365	3.9	90.1	9.65	9.86	9.35	11.40	2.05
8	Sterling Kingsize Dual	Value	3517	3.2	90.5	9.00	9.21	8.50	10.99	2.49
9	Carlton Superking Red	Subvalue	3327	3.0	77.4	7.75	7.99	7.50	9.53	2.03
10	Players Kingsize Crushball	Subvalue	2961	2.7	86.9	7.65	7.91	7.29	9.65	2.36
Total top 10			63 543	57.3	–	–	–	7.29	11.65	4.36
17	Regal Kingsize Blue	Premium	1348	1.2	226	82.48	10.90	10.20	13.25	3.05
18	Marlboro Gold Kingsize	Premium	1332	1.2	198	72.26	10.70	10.44	12.71	2.27
RYO 30 g										
1	Amber Leaf	Midprice/ premium	2887	31.2	92.7	13.00	13.18	12.20	14.99	2.79
2	Gold Leaf & Papers	Value	2298	24.8	89.8	10.90	10.73	9.99	15.25	5.26
3	Golden Virginia Original & Papers	Midprice/ premium	1041	11.3	86.9	13.35	13.19	12.50	15.99	3.49
Total top 3			6226	67.3	–	–	–	9.99	15.99	5.00

*Kensitas Club, a Scottish brand manufactured by Gallaher Group, a subsidiary of Japan Tobacco, was relaunched in UK in February 2018. At this time, a new Superking variant was introduced and the RRP for the established Kingsize variant was vastly reduced by the manufacturers, from £10.55 to £7.65. The RRP of £10.55 suggested for Kensitas Club Kingsize by the wholesalers in April 2018 reflects the pricing of the brand before the relaunch.

†Wholesaler RSP figures for cigarettes from April 2018 and for RYO from September 2018.

RRP, recommended retail price; RSP, recommended sales price; RYO, roll your own.

Multivariable models of all sales of packets of 20 cigarettes indicated a relationship between purchase price and area deprivation, with prices paid 50 pence (5.6%, p<0.001) lower in the highest deprivation quintile and 56 pence (6.3%, p<0.001) lower in the second highest quintile of deprivation relative to the lowest deprivation areas (table 3). We did not observe the expected negative gradient with retailer density. The mean price of total sales was 10 pence lower in neighbourhoods with the second highest outlet density compared with areas with the lowest density. While prices were 4 pence higher in shops in areas with the highest density, this difference was not statistically significant. Relative to large urban areas, mean prices were significantly lower in rural areas (17 pence; 2.0%, p=0.004). For the price of the lowest priced cigarette brand sold, there was no relationship with income deprivation, retail outlet density or urban/rural status (result not shown in table).

**Table 3:** Cigarette and RYO regression models

Dependent variable		Cigarette packets of 20										RYO 30 g					
		Sales price		Brand variant segment								Sales price		Lowest priced brand variant		Brand variant segment	
				Subvalue		Value		Midprice		Premium		Value					
N		110 830		110 830		110 830		110 830		110 830		9248		270		9248	
Coefficients		Unstandardised coefficients (£)	Sig.	OR	Sig.	OR	Sig.	OR	Sig.	OR	Sig.	Unstandardised coefficients (£)	Sig.	Unstandardised coefficients (£)	Sig.	OR	Sig.
Constant		8.99	<0.001	0.51	<0.001	0.58	<0.001	0.23	<0.001	0.143	<0.001	12.40	<0.001	10.85	<0.001	0.61	0.002
Income	1 lowest	–	–	–	–	–	–	–	–	–	–	–	–	–	–	–	–
Deprivation	2	−0.20	0.020	1.15	0.140	1.02	0.799	0.99	0.872	0.66	0.006	−0.03	0.540	−0.09	0.437	0.98	0.910
Quintile	3	−0.40	<0.001	1.54	<0.001	0.90	0.191	0.86	0.073	0.41	<0.001	−0.18	<0.001	−0.09	0.457	1.05	0.722
	4	−0.56	<0.001	2.15	<0.001	0.74	<0.001	0.66	<0.001	0.28	<0.001	−0.49	<0.001	−0.09	0.453	1.73	<0.001
	5 highest	−0.50	<0.001	2.15	<0.001	0.73	<0.001	0.72	<0.001	0.23	<0.001	−0.34	<0.001	−0.23	0.052	1.66	<0.001
Outlet	1 lowest	–	–	–	–	–	–	–	–	–	–	–	–	–	–	–	–
Density	2	−0.14	0.008	1.26	0.012	0.86	0.082	0.90	0.214	0.77	0.085	−0.11	0.027	−0.23	0.063	1.13	0.394
Quintile	3	−0.08	0.102	1.19	0.034	0.87	0.075	0.91	0.252	0.94	0.713	0.06	0.208	−0.12	0.338	0.98	0.914
	4	−0.10	0.050	1.25	0.013	0.83	0.030	0.89	0.202	1.00	0.976	−0.10	0.041	−0.28	0.030	1.13	0.406
	5 highest	0.04	0.591	1.14	0.162	0.89	0.159	0.89	0.175	1.13	0.400	0.09	0.074	−0.21	0.109	1.05	0.767
Urban/rural	Large urban	–	–	–	–	–	–	–	–	–	–	–	–	–	–	–	–
Status	Other urban	−0.09	0.045	1.17	0.007	0.91	0.115	0.95	0.368	0.73	0.003	−0.07	0.033	−0.09	0.324	1.09	0.373
	Small towns	−0.05	0.430	0.95	0.658	1.04	0.717	1.12	0.282	0.81	0.279	0.15	0.003	0.05	0.735	0.66	0.024
	Rural areas	−0.17	0.004	1.26	0.021	0.80	0.013	1.00	0.980	0.81	0.178	0.02	0.637	0.10	0.422	0.73	0.031

RYO, roll your own.

Models of the prices of all RYO 30 g packs sold were similar to those for cigarettes (table 3); prices paid were 34 pence (2.7%, p<0.001) lower in the highest deprivation quintile and 49 pence (4.0%, p<0.001) lower in the second most deprived quintile relative to the least deprived quintile. Prices of total sales were also lower in areas with the fourth highest level of outlet density (11 pence; 0.9%, p=0.027) and the second highest outlet density (10 pence; 0.8%, p=0.041). The lowest priced RYO product sold was 28 pence (2.5%) cheaper in areas with the second highest level of outlet density. There was no relationship between lowest priced RYO brand sold and income deprivation or urban/rural status.

### The proportions of cigarette brand and RYO brand variants sold by price segments

**Figure 1:**
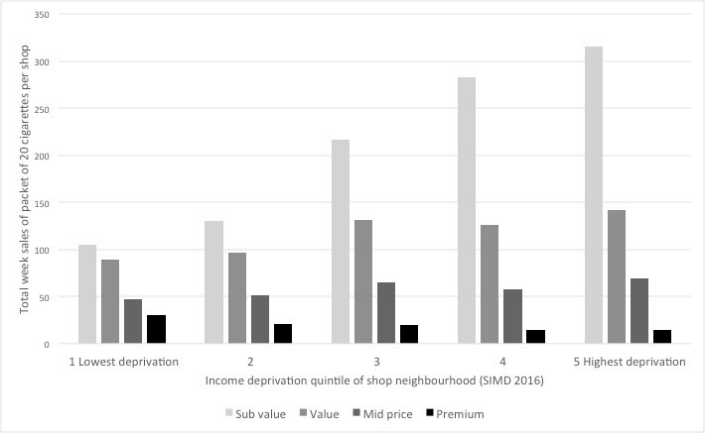
Cigarette segments by area level income deprivation. SIMD, Scottish Index of Multiple Deprivation.

Stores sold a wide selection of cigarette brands, with an average of 40.9 brands per store in the study week, ranging from 2 brands to 78 brands sold. Among the shops, 93.8% sold at least one brand from each of the four price segments. The most popular segment was subvalue, with 51.8% of tobacco sales, followed by value (28.9%), midprice (14.4%) and premium (5.0%) (table 1). In stores in the most deprived areas, the proportion of total sales that were subvalue was 58.3%, compared with 38.6% in stores in the lowest deprivation areas. The equivalent figures for premium brand variants were 2.7% and 11.1%, respectively. At each level of deprivation sales of subvalue cigarettes were dominant, followed by value, midprice and premium (figure 1). Multivariate models of the relationship indicated that the odds that a brand variant sold was subvalue was 2.15 (OR p<0.001) in shops in the most deprived areas relative to those in the least deprived, while the equivalent OR for premium brands was 0.23 (p<0.001) (table 3).

For RYO, two price segments were explored, value and midprice/premium, with sales divided between them at 44.5% and 55.3%, respectively (table 1). In stores in the most deprived areas, the proportion of sales that were value brands was 51.9% compared with 37.0% in those in the most affluent areas. For midvalue/premium this ranged from 48.1% in the most deprived to 62.5% in the most affluent. Multivariate models suggest significantly higher odds of value brand RYO purchases in the top two deprived quintiles (OR 1.73 quintile 4 and OR 1.66 quintile 5, both p<0.001) (table 3).

### The price of individual brands of cigarettes and RYO

Descriptive data outlines the characteristics of the best selling cigarette (packets of 20) brand variants in the study week (table 2). Of the top 10 brands, 5 were subvalue, 3 value and 2 midprice. Most shops stocked a broad range of the most popular brands with 6 of the top 10 biggest selling cigarette brand variants sold in more than 90% of the study shops. The prices at which shops sold products varied significantly with a range of £2.03 or greater for all the top 10 brands.

Multivariate models of shop prices of these 10 brands and the top two premium brands indicate that most had lower prices in the fourth and fifth most deprived quintiles relative to the least deprived quintile, but these differences were small and insignificant, with the exception of models for some higher priced brands (table 4). One premium brand, Regal Kingsize Blue, was significantly cheaper, by 17 pence and 13 pence, in the fourth and fifth most deprived quintiles. A further premium brand, Marlboro Gold Kingsize, was 17 pence cheaper and a midprice brand, Mayfair Kingsize Original Blue, was 12 pence cheaper in stores in stores located in the fourth most deprived quintile.

**Table 4:** Cigarette brand variant price models: shop prices of top selling brands of packet of 20 cigarettes

Dependent variable		Cigarette packet of 20: brand variant name											
		Players Kingsize Real Red	Players Superking Real Red	L&B Kingsize Blue Real Blue	Kensitas Club Kingsize	JPS Kingsize Real Blue	L&B Kingsize Original Silver	Mayfair Kingsize Original Blue	Sterling Kingsize Dual	Carlton Superking Red	Players Kingsize Crushball	Regal Kingsize Blue	Marlboro Gold Kingsize
Price segment		Subvalue	Subvalue	Value	Subvalue	Value	Midprice	Midprice	Value	Subvalue	Subvalue	Premium	Premium
Sales rank		1	2	3	4	5	6	7	8	9	10	17	18
Shop N		266	260	220	177	260	255	247	248	212	238	226	198
Unstandardised coefficients (£)													
**Constant**		7.97*	7.95*	8.37*	7.98*	8.70*	9.75*	9.98*	9.29*	8.08*	7.98*	11.36*	11.09*
**Income**	1 lowest	–	–	–	–	–	–	–	–	–	–	–	–
**Deprivation**	2	0.03	0.03	−0.01	−0.04	0.01	−0.02	−0.05	−0.01	0.00	−0.05	−0.11	0.02
**Quintile**	3	−0.05	−0.04	−0.07	−0.03	−0.05	−0.06	−0.10	−0.12*	−0.06	−0.07	−0.09	−0.10
	4	−0.05	−0.05	−0.13	−0.06	−0.08	−0.05	−0.12*	−0.09	−0.05	−0.08	−0.17*	−0.17*
	5 highest	−0.02	0.00	−0.07	−0.04	0.01	−0.03	−0.09	−0.08	−0.01	−0.03	−0.13*	−0.07
**Outlet**	1 lowest	–	–	–	–	–	–	–	–	–	–	–	–
**Density**	2	−0.05	−0.06	−0.03	−0.06	−0.08	−0.03	−0.08	−0.05	−0.05	−0.04	−0.04	−0.10
**Quintile**	3	−0.02	−0.01	−0.03	−0.04	−0.04	0.00	−0.05	−0.01	−0.03	0.02	−0.06	0.01
	4	−0.06	−0.06	−0.14*	−0.02	−0.09	−0.09	−0.07	−0.02	−0.07	−0.04	−0.01	−0.09
	5 highest	0.00	0.00	0.01	−0.01	−0.02	−0.01	0.03	0.09	−0.01	0.04	0.06	0.09
**Urban/rural**	Large urban	–	–	–	–	–	–	–	–	–	–	–	–
**Status**	Other urban areas	−0.04	−0.03	−0.06	−0.08	−0.02	−0.02	0.01	0.01	−0.01	−0.01	0.01	0.02
	Small towns	−0.05	−0.05	−0.11	−0.18*	−0.04	−0.13	−0.02	−0.06	−0.06	−0.02	−0.05	−0.14
	Rural areas	−0.12*	−0.13*	−0.17*	−0.21*	−0.17*	−0.17*	−0.06	−0.13*	−0.16*	−0.08	−0.12	−0.12

*P value of coefficient significant at 0.05 level.

Models showed that most brand variants had lower prices in areas with medium levels of outlet density (quintiles 2–4), but these relationships were mostly non-significant (with the exception of one value brand that was 14 pence cheaper in the stores in areas with the second highest levels of density). The strongest and most consistent relationship with brand price was found for urban/rural status; 8 of the 12 cigarette brand variants assessed were significantly cheaper (12–21 pence) in rural areas relative to large urban areas.

Three RYO brands dominated sales (Amber Leaf, Gold Leaf & Papers and Golden Virginia Original & Papers), totalling 67.3% of all RYO sales (table 2). During the course of the week, each of these brands was sold in over 86% of stores. Like cigarette packets, the prices varied significantly, with price differences ranging by £2.79, £5.26 and £3.49 for these three brands. Regression analysis of the relationship between area characteristics and prices of these three brands finds that Amber Leaf, a midprice/premium brand variant, was 15 pence cheaper in the most deprived relative to least deprived quintile and 16 pence cheaper in small towns relative to large urban areas (online supplementary table 1). Golden Virginia Original and Papers, another midprice/premium product, was 24 pence cheaper in rural areas relative to large urban areas. There was no relationship between RYO brand price variation and tobacco outlet density.

## Discussion

This study assessed how tobacco price structures in small convenience stores in Scotland vary geographically by area-level deprivation, urban/rural status and tobacco retail outlet density. The key findings show that the price paid for tobacco is lower in more deprived areas for both cigarettes and RYO compared with more affluent areas and that the price paid is also lower in rural areas compared with urban areas. Furthermore, we have shown that shops sell a wide range of brands but that subvalue brands are dominant in all areas, considerably more so in areas of high deprivation, and for some individual brands price varies by deprivation and rurality, with the cheapest products found in the most deprived or rural areas. Our results confirm that the dominance of subvalue brands, particularly in more deprived areas, is the driving force behind the difference in price paid for tobacco between neighbourhoods, rather than retailers discounting particular brands. This highlights the importance of ‘cheap’ tobacco products to the consumer and the market. Contrary to suggestion that price competition is one pathway that connects outlet density and smoking behaviours, this analysis found limited evidence of a relationship between tobacco retail density and lower prices.

Although we found some price variation in individual brands, the key difference in price paid for tobacco in different kinds of area was largely driven by the division of sales in each of the price segments. The dominance of subvalue brands in all areas, but most especially so in the most deprived areas, provides further evidence of the price sensitivity of consumers.^1^ With smoking rates highest in the most deprived areas, the results suggest that the availability of cheap tobacco may help tobacco companies to retain price sensitive consumers that in turn contributes to health inequalities. Recent evidence from the UK suggests that the price of subvalue cigarettes has remained relatively unchanged over the past 20 years in spite of numerous tax increases.^9^ The tobacco industry has strategically worked to absorb these increases by incentivising stores to stock a wide range of brands^31 32^ allowing tobacco companies to segment the market by price. This segmentation enables the industry to maintain low prices on the subvalue brands in part through processes of both overshifting and undershifting taxes.^9^ This means that tobacco companies increase the price of premium brands to absorb tax rises on the cheapest brands, protecting their more price sensitive customers. Our results confirm that the market share of premium brands decreases and subvalue brands increase, in line with increasing area-level deprivation. These findings confirm self-report and survey-based estimates of price differentiation by area-level deprivation elsewhere,^23 33^ but importantly, these results are based on a robust point-of-sale database covering all sales, rather than survey or self-report data. These findings also support previous international literature based on tobacco industry documents that emphasised the ‘aggressive pricing strategies’ that enabled the emergence of discounted brands, their dominance of the market and their contribution to slowing the earlier decline in smoking rates.^34^

This study has several strengths including being the first to assess the relationship between neighbourhood characteristics and tobacco price using comprehensive store-level information on tobacco sales. The results may however be limited in that the data were restricted to convenience stores and studies comparing retailer types have found that tobacco price varies by retailer type and that the relationship between price and area-level characteristics may do also.^12^ Our analysis did not find a strong or consistent association between tobacco retail outlet density and price. There may be numerous reasons for this, including the possibility that no such relationship exists, but it may also be due to measurement issues, including collinearity between the area variables in the analysis. This analysis found cigarette and RYO brands were more expensive in large urban areas. The high costs of operating retail businesses in premium city retail sites may obscure the effects of price competition. Our measure of outlet density also may not be a precise indicator of retail competition experienced by the convenience store retailers because it included all retail types, while price competition may be primarily between similar retailer types. Future research could include price data for all retail types.

Despite these limitations this study has important findings that add to current policy discussions regarding tobacco retail interventions, both in Scotland and internationally. One potential policy response to tobacco industry brand segmentation is to broaden future price interventions beyond product duties. A combination of both MUP and a price cap at the upper end would limit the ability of the industry to absorb tax rises and segment the market, thus deterring the continuing supply of subvalue brands. Tobacco MUP has already been implemented elsewhere, and in New York, the minimum price per cigarette pack has been set at $13, the highest price in the USA.^35^ In 2017, a minimum excise tax (MET) (equivalent to £5.60 per pack of 20 cigarettes) was introduced in the UK aiming to increase the price of cheap cigarettes. While such an intervention may narrow the price gap between the market segments, it does not prevent the price shifting mentioned earlier and as such the cheapest brands may shift closer to the MET, with prices increasing in the premium brands.^26^ MUP is therefore an alternative that would limit this price shifting. Examining the potential effect of minimum pricing of tobacco in the USA research concluded that a national minimum price strategy would have a significant impact on sales and significant benefits for public health.^36^

With growing international interest in the ‘Tobacco Endgame’, policymakers should identify measures that counter industry tactics enabling the continued sales of cheap tobacco. Any increase in the price of tobacco will have the greatest effect on lower income groups, those for whom smoking rates are highest. There are opportunities to learn from the strategies adopted elsewhere, particularly in the area of alcohol policy. In 2018, Scotland became the first country to implement a minimum price for alcohol, but many argued that the implementation of MUP was a regressive policy, impacting the most on the poorest. Similar arguments could be made with a MUP for tobacco. Our analysis shows that any increase in the price of subvalue tobacco will impact the majority of smokers, but it will have the greatest impact on those in the poorest neighbourhoods. These areas also have the highest smoking rates and as such may experience the greatest gains in health as a result of successful quit attempts. To achieve the ‘Tobacco Endgame’, policies must address the entire population *and* those most affected by harm.

**What this paper adds**

- Previous analysis of tobacco price and neighbourhood-level characteristics suggests that the price smokers pay varies between areas. This research has been limited by pricing data reliant on self-report surveys or store audits.
- This study is the first neighbourhood analysis of cigarette and roll-your-own (RYO) tobacco price to use comprehensive sales data describing all tobacco products purchased over a 1-week period, supporting a price analysis of brand segments and individual brands with neighbourhood-level income deprivation, tobacco retail outlet density and rural/urban status.
- There is a strong relationship between price paid and income deprivation. The average price paid for cigarettes and RYO tobacco was lower in the most deprived areas: 50 pence (5.6%) lower for cigarettes and 34 pence (2.7%) lower for RYO tobacco.
- The lower prices paid in more deprived neighbourhoods primarily reflects greater sales of cheap brands in these areas, rather than retailers reducing the prices of individual brands.
- We report limited evidence of a relationship between price paid for tobacco products and tobacco retail outlet density.
