# Supplementary material for: Using point-of-sale data to examine tobacco pricing across neighbourhoods in Scotland
Source: Tob Control. 2020 Mar 19;30(2):168–76. doi: 10.1136/tobaccocontrol-2019-055484 (PMC7907555; doi:10.1136/tobaccocontrol-2019-055484)
Supplement: Supplementary data [file tobaccocontrol-2019-055484supp001.pdf]

Supplementary Table 1: RYO brand variant price models – shop prices of top selling brands of RYO Packets

		**RYO 30g: Brand variant name**		
**Dependent variable**		**Amber Leaf**	**Gold Leaf and Papers**	**Golden Virginia Original and Papers**
Price segment		mid price/premium	value	mid price/premium
Sales rank		1	2	3
Shop N		254	246	238
Unstandardized coefficients (£)				
**Constant**		13.32*	10.85*	13.34*
**Income deprivation quintile**	1 lowest	-	-	-
	2	-0.08	-0.04	-0.01
	3	-0.12	-0.01	-0.08
	4	-0.13	-0.08	-0.09
	5 highest	-0.15*	-0.09	-0.04
**Outlet density quintile**	1 lowest	-	-	-
	2	-0.02	-0.01	-0.09
	3	0.03	0.03	-0.04
	4	-0.04	-0.13	-0.06
	5 highest	0.07	0.07	0.02
**Urban/rural status**	Large urban	-	-	-
	Other urban areas	-0.06	-0.06	-0.06
	Small towns	-0.16*	-0.19	-0.14
	Rural areas	-0.13	-0.15	-0.24*

*P value of coefficient signficant at 0.05 level
